# Celastrol Attenuates the Invasion and Migration and Augments the Anticancer Effects of Bortezomib in a Xenograft Mouse Model of Multiple Myeloma

**DOI:** 10.3389/fphar.2018.00365

**Published:** 2018-05-03

**Authors:** Muthu K. Shanmugam, Kwang S. Ahn, Jong H. Lee, Radhamani Kannaiyan, Nurulhuda Mustafa, Kanjoormana A. Manu, Kodappully S. Siveen, Gautam Sethi, Wee J. Chng, Alan P. Kumar

**Affiliations:** ^1^Department of Pharmacology, Yong Loo Lin School of Medicine, National University of Singapore, Singapore, Singapore; ^2^College of Korean Medicine, Kyung Hee University, Seoul, South Korea; ^3^Cancer Science Institute of Singapore, Centre for Translational Medicine, Singapore, Singapore; ^4^Department of Hematology-Oncology, National University Cancer Institute, Singapore, National University Health System, Singapore, Singapore; ^5^Medical Sciences Cluster, Yong Loo Lin School of Medicine, National University of Singapore, Singapore, Singapore; ^6^Curtin Medical School, Faculty of Health Sciences, Curtin University, Perth, WA, Australia; ^7^National University Cancer Institute, Singapore, National University Health System, Singapore, Singapore

**Keywords:** celastrol, bortezomib, NF-κB, multiple myeloma, xenograft models

## Abstract

Several lines of evidence have demonstrated that deregulated activation of NF-κB plays a pivotal role in the initiation and progression of a variety of cancers including multiple myeloma (MM). Therefore, novel molecules that can effectively suppress deregulated NF-κB upregulation can potentially reduce MM growth. In this study, the effect of celastrol (CSL) on patient derived CD138+ MM cell proliferation, apoptosis, cell invasion, and migration was investigated. In addition, we studied whether CSL can potentiate the apoptotic effect of bortezomib, a proteasome inhibitor in MM cells and in a xenograft mouse model. We found that CSL significantly reduced cell proliferation and enhanced apoptosis when used in combination with bortezomib and upregulated caspase-3 in these cells. CSL also inhibited invasion and migration of MM cells through the suppression of constitutive NF-κB activation and expression of downstream gene products such as CXCR4 and MMP-9. Moreover, CSL when administered either alone or in combination with bortezomib inhibited MM tumor growth and decreased serum IL-6 and TNF-α levels. Overall, our results suggest that CSL can abrogate MM growth both *in vitro* and *in vivo* and may serve as a useful pharmacological agent for the treatment of myeloma and other hematological malignancies.

## Introduction

Multiple myeloma (MM) is a plasma cell proliferative disorder with distinct genomic instability, reflected by its heterogeneous clinical course. MM is the second most common hematological malignancy after non-Hodgkin’s lymphoma (Hideshima et al., 2004; Raab et al., 2009; Anderson and Carrasco, 2011; Agarwal and Ghobrial, 2013). In newly diagnosed disease, high-dose chemotherapy followed by autologous hematopoietic stem-cell transplantation provides a moderate survival benefit (Richardson et al., 2005; Abdi et al., 2013). Advent of novel targeted therapies has expanded the armamentarium of MM therapy thereby resulting in increased longevity (Palumbo and Cavallo, 2012). Bortezomib, a small molecule inhibitor of proteasome is an important addition to the chemotherapy of myeloma (Cavo, 2006; Moreau et al., 2012; Suzuki, 2013). However, side effects and development of chemoresistance remains as the limiting factor in the usage of such drugs. Combination strategies have been exploited in cancer chemotherapy for long to gain the advantage of improved efficacy with fewer side effects (Tu et al., 1998; Roodman, 2002; Ackler et al., 2010; Galmarini et al., 2012; Palumbo and Cavallo, 2012).

It has been reported that potential interactions between myeloma cells and the bone marrow microenvironment plays an important part in the progression of disease (Chauhan et al., 1996; Roodman, 2002). For example, transcription factor, NF-κB induced adhesion of myeloma cells to the bone marrow stromal cells promotes the secretion of IL-6 by the stromal cells. IL-6 in turn acts on the myeloma cells in paracrine manner to activate the STAT3 signaling cascade (Chauhan et al., 1996; Ihle, 1996; Catlett-Falcone et al., 1999; Puthier et al., 1999a; Barille et al., 2000). Thus, both NF-κB and STAT3 transcription factors have been reported to be involved in myelomagenesis, survival, invasion, migration, chemoresistance, and osteolysis in MM (Barille et al., 2000; Ni et al., 2001; Bharti et al., 2004; Van de Donk et al., 2005; Li and Sethi, 2010; Salem et al., 2012; Shanmugam et al., 2013; Chai et al., 2015; Li et al., 2015).

STAT3 has also been reported to upregulate the expression of anti-apoptotic proteins like Bcl-2, Bcl-xL, and Mcl-1 in myeloma cell lines (Tu et al., 1998; Puthier et al., 1999a,b; Spets et al., 2002; Quintanilla-Martinez et al., 2003; van de Donk et al., 2003; Ackler et al., 2010). Constitutive expression of STAT3 provides survival advantage to MM cells (Catlett-Falcone et al., 1999) and approximately 48% of patient samples display constitutively activated STAT3 (Quintanilla-Martinez et al., 2003). Moreover 15–20% of myeloma samples also display activating mutations in NF-κB pathway (Ni et al., 2001; Bharti et al., 2004; Annunziata et al., 2007; Keats et al., 2007; Demchenko et al., 2010).

Alternative and complimentary therapies are gaining increased attention in cancer treatment for their potential to compliment the antitumor effects of both chemotherapy and targeted agents (Shanmugam et al., 2011, 2016, 2017; Yang et al., 2013; Tang et al., 2014; Hsieh et al., 2015; Bishayee and Sethi, 2016). Celastrol (CSL) is a quinine methide triterpenoid compound derived from traditional Chinese medicinal plant *Tripterygium wilfordii*. The extracts of this plant has been used for years in Chinese traditional medicine for its anti-inflammatory properties (Tao and Lipsky, 2000; Kannaiyan et al., 2011a,b,c). Recently there has been a tremendous increase in the interest about the potential anticancer properties of CSL (Su et al., 2009; Shanmugam et al., 2016). It has been reported to display anti-inflammatory and anti-neoplastic effects in various tumor cells including MM via modulation of diverse oncogenic molecular targets (Kannaiyan et al., 2011a,c).

Our group has reported previously that CSL can induce apoptosis on its own and can enhance the apoptotic effects of both bortezomib and thalidomide in MM cell lines (Kannaiyan et al., 2011a). In the current study, we provide evidence to indicate that CSL alone can decrease the tumor growth and can also augment the antitumor effect of bortezomib in mouse model. CSL treatment in combination with bortezomib was found to downregulate the NF-κB regulated prosurvival and anti-apoptotic genes, which might be one of the probable mechanism(s) though which CSL potentiates the action of bortezomib. Moreover, CSL can also inhibit the invasion and migration of cultured myeloma cells *in vitro* and downregulate the expression of CXCR4 and MMP-9 proteins.

## Materials and Methods

### Reagents

Celastrol with purity greater than 98% was purchased from Alexis Biochemicals (San Diego, CA, United States). RPMI 1640, 0.4% trypan blue vital stain, and antibiotic-antimycotic mixture were obtained from Invitrogen (Carlsbad, CA, United States). Propidium iodide (PI), MTT, Tris, glycine, NaCl, SDS, BSA, and β-actin antibody (mouse monoclonal) was obtained from Sigma-Aldrich Chemical Co. (St. Louis, MO, United States). FBS was purchased from BioWest (Miami, FL, United States). Bortezomib (Velcade, PS341) was purchased from LC Laboratories (Woburn, MA, United States). Nuclear extraction and DNA binding kits was obtained from Active Motif (Carlsbad, CA, United States). Caspase-Glo 3/7 Assay kit was purchased from Promega (Madison, WI, United States). Rabbit polyclonal antibodies against Poly(ADP-ribose) polymerase (PARP), MMP-9, caspase-3, and goat anti-rabbit-horse radish peroxidase (HRP) conjugate and goat anti-mouse HRP conjugate were purchased from Santa Cruz Biotechnology (Santa Cruz, CA, United States). Rabbit polyclonal antibody against CXCR4 was purchased from Abcam (Cambridge, MA, United States). CXCL12 was purchased from ProSpec-Tany TechnoGene Ltd. (Rehovot, Israel). ELISA kits for mouse IL-6 and TNF-α were purchased from R&D Systems Inc. (Minneapolis, MN, United States).

### Cell Lines and Culture Conditions

The human MM cell lines U266, H929, and KMS11 were kindly provided by Dr. Leif Bergsagel from Mayo Clinic, Scottsdale, AZ, United States. H929 cells were cultured in RPMI 1640 medium containing 2-mercaptoethanol at a final concentration of 0.05 mM, supplemented with 10% FBS. The other two MM cell lines were cultured in RPMI 1640 medium containing 1× antibiotic-antimycotic with 10% FBS. These MM cells were used to analyze the effect of CSL alone and/or in combination with bortezomib on viability, invasion, migration, and apoptosis.

Primary MM patient cells were obtained from bone marrow aspirates of patients after obtaining informed written consent and with ethical approval from the NUS Institutional Review Board. Peripheral blood mononuclear cells were separated with RBC lysis buffer and subsequently CD138^+^ plasma cells were isolated using magnetic cell sorting with CD138 EasySep magnetic nanoparticles (Stemcell Technologies, Singapore) according to manufacturer’s instructions. Purified CD138^+^ patient cells were grown in IMDM, GlutaMAX (Gibco, Invitrogen), supplemented with 20% FBS, 100 U/mL penicillin and 100 μg/mL streptomycin, 10 ng/mL of IL-6 (Miltenyi Biotec, Surrey, United Kingdom) and 100 ng/mL of rhIGF-1(R&D Systems, Oxford, United Kingdom). All cells were grown at 37°C in a humidified atmosphere with 5% CO_2_.

### Cell Proliferation Assay

The anti-proliferative effect of CSL against CD138^+^ cells from patient samples was determined by the MTT dye uptake method as described previously (Siveen et al., 2014).

### Flow Cytometric Analysis

The apoptotic effect of CSL against CD138^+^ cells from patient samples was determined by flow cytometric analysis using PI staining. Briefly the cells were incubated with 0, 1, 2.5, and 5 μM CSL for 72 h at 37°C. Thereafter the cells were washed, fixed with 70% ethanol, and incubated for 30 min at 37°C with 0.1% RNase A in PBS. Cells were washed again, resuspended, and stained with PBS containing 25 μg/mL PI for 30 min at room temperature. Cell distribution across the cell cycle was analyzed with a CyAn ADP flow cytometer (DakoCytomation).

### Migration Assay

U266 cells (50 × 10^4^/well) were plated in 300 μl cell culture media with and without 1 μM CSL in the top chambers of 24-well transwell inserts with 8 μm pores (Greiner Bio-One ThinCert^TM^, Monroe, NC, United States). Cell culture medium (600 μl) containing the Recombinant Human B-cell chemoattractant, CXCL12 (100 ng/ml; ProSpec, Ness-Ziona, Israel) was added to the bottom chamber and incubated for 12 h. Thereafter, the migration assay was performed as described previously (Siveen et al., 2014).

### Invasion Assay

An invasion assay was performed with U266 cells in 24-well plates with ThinCert tissue culture insert containing polycarbonate membranes (8 μm pore size) as described previously (Siveen et al., 2014).

### Western Blotting

For detection of various proteins, celastrol and/or bortezomib treated whole-cell extracts were lysed in lysis buffer [20 mM Tris (pH 7.4), 250 mM NaCl, 2 mM EDTA (pH 8.0), 0.1% Triton X-100, 0.01 mg/ml aprotinin, 0.005 mg/ml leupeptin, 0.4 mM PMSF, and 4 mM NaVO_4_] and western blot assay was carried out as described previously (Baek et al., 2017). The densitometric analysis of the scanned blots was done with Image J software and the results are expressed as fold change relative to the control.

### Caspase-3 Colorimetric Assay

Caspase-3 activity was measured using a Caspase-Glo 3/7 Assay (Promega, Madison, WI, United States) kit as described previously (Ningegowda et al., 2017).

### NF-κB DNA Binding Assay

To determine NF-κB activation, we performed DNA binding assay using TransAM NF-κB Kit from Active Motif, as described previously (Siveen et al., 2014).

### Apoptosis Detection by ELISA

Apoptosis of cells was also determined using the Cell Death Detection ELISA^PLUS^ Kit (Roche Diagnostics, Indianapolis, IN, United States) as described previously (Dai et al., 2015).

### Xenograft MM Mouse Model

All the procedures involving animals were reviewed and approved by National University of Singapore Institutional Animal Care and Use Committee. Male athymic balb/c nude mice (BRC, Biopolis, Singapore) were implanted with 2 × 10^6^ cells with Human MM U266 cell lines subcutaneously. When tumors have reached more than 0.3 cm in diameter, the mice were randomized into four groups. Group I (control) received corn oil 100 μl i.p. for 5 days a week, group II received 0.25 mg/kg celastrol in 100 μl corn oil for 5 days a week, group III received 0.25 mg/kg bortezomib in 100 μl corn oil i.p. weekly and group IV received 0.25 mg/kg celastrol in 100 μl corn oil i.p. 5 days a week and 0.25 mg/kg bortezomib in 100 μl corn oil i.p. weekly for three consecutive weeks. The tumor volume and body weight of the mice were monitored twice a week for the duration of the experiment. On completion of the treatment period, mice were euthanized by i.p. phentobarbital (40 mg/kg b.w) followed by cervical dislocation, blood collected by heart puncture and then tumors were dissected and diameters measured. The tumor volume was calculated using the formula [*L* ×*W*^2^]/2, where *W* and *L* are the width (short diameter) and the length (long diameter) of the tumor. Half of the tumor tissue was fixed in formalin and embedded in paraffin for immunohistochemistry staining. The remaining tumor tissue was snap frozen in liquid nitrogen and stored at -80°C.

### Serum Levels of IL-6 and TNF-α in MM Tumor Bearing Mice

The serum levels of IL-6 and TNF-α were determined using ELISA kits (R&D systems) as described previously (Siveen et al., 2014).

### Statistical Analysis

Data are expressed as the mean ± SD. In all figures, vertical error bars denote the SD. The significance of differences between groups was evaluated by Student’s *t*-test and one way analysis of variance, (ANOVA) test. A “*p*” value of less than 0.05 was considered statistically significant.

## Results

**FIGURE 1:**
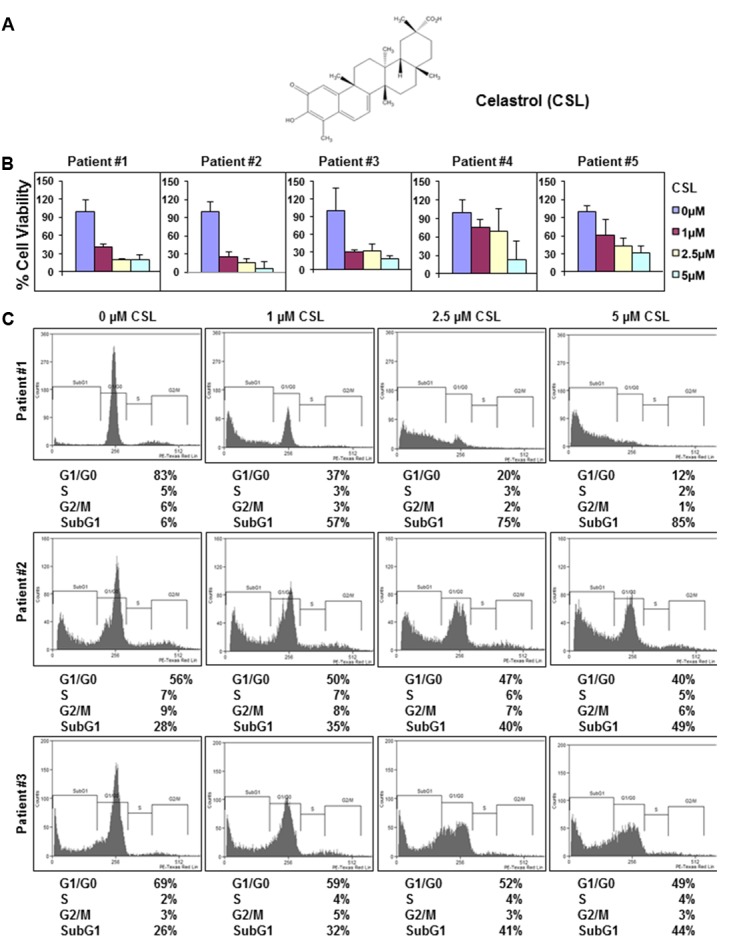
Celastrol (CSL) suppresses the proliferation of CD138+ cells derived from MM patients. **(A)** Chemical structure of CSL. **(B)** CD138+ cells were isolated from MM patient samples as described in the section “Materials and Methods.” The cells were plated in triplicate, treated with 0, 1, 2.5, and 5 μM CSL for 72 h and then subjected to MTT assay to analyze proliferation of cells. Columns, mean; bars, SD. **(C)** CD138+ cells from MM patient samples were plated, thereafter treated with 0, 1, 2.5, and 5 μM CSL for 72 h and then washed, fixed, stained with PI, and analyzed for DNA content by flow cytometry.

In this study, we primarily investigated whether CSL (chemical structure shown in **Figure 1A**) can potentiate the apoptotic effect of bortezomib in MM cells and a xenograft mouse model.

### Anti-proliferative Effects of CSL in Patient Samples

Our prior published report has suggested that celastrol can significantly inhibit the proliferation of diverse MM cell lines (Kannaiyan et al., 2011a). Hence, the anti-myeloma effect of CSL was now evaluated by examining its potential effects on the viability of CD138^+^ cells derived from 5 MM patients. The cells were treated with different doses of CSL for 72 h and then examined for cell proliferation by MTT method. The data obtained indicated that CSL can significantly suppress the proliferation of CD138^+^ cells in a concentration-dependent manner (**Figure 1B**). We next investigated whether treatment with CSL can induce apoptosis in CD138^+^ cells using flow cytometric analysis. We focused on CD138^+^ cells derived from patients 1–3 since they showed better effects on cell viability. The result shows that CSL can indeed increase the accumulation of the treated cells in the Sub-G1 phase, which is a marker of apoptosis, in a dose-dependent manner (**Figure 1C**).

### Celastrol Inhibits the Invasion and Migration of MM Cells

**FIGURE 2:**
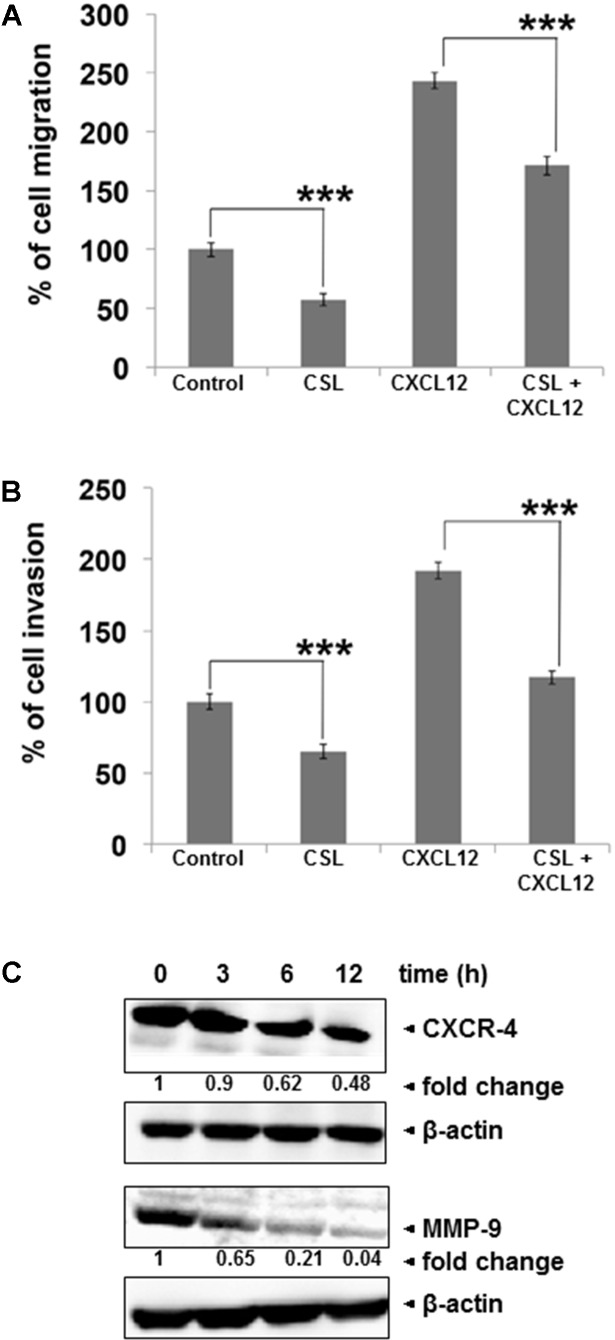
Celastrol inhibits migration and invasion of MM cell lines. **(A)** U266 cells (50 × 10^4^/well) in cell culture media with and without 1 μM CSL was added to the top chambers of 24-well transwell inserts with 8-μm pores. Cell culture medium (600 μl) containing the recombinant human B-cell chemoattractant, CXCL12 was added to the bottom chamber and incubated for 12 h. After incubation, the transwell migration toward CXCL12 was measured using calcein-AM (5 μM) staining and measuring the fluorescence intensity. Data are expressed as percentage of cell migration. Columns, mean; bars, SD. ^∗∗∗^*p* < 0.001. **(B)** U266 cells (50 × 10^4^) in suspension were starved in serum-free RPMI-1640 for 3 h, and then loaded onto the Matrigel-coated inserts in the upper chambers of tissue culture inserts placed in 24 well plates. The wells of the plate were filled with 600-μl of 10% FBS-containing cell culture media with 100 ng/ml CXCL12. CSL (1 μM) was added with the cells to the upper chamber. Plates were then incubated at 37°C for 12 h. Matrigel invasion toward CXCL12 was measured by staining the cells with calcein-AM (5 μM) and measuring the fluorescence intensity. Data are expressed as percentage of mean cell invasion. Columns, mean; bars, SD. ^∗∗∗^*p* < 0.001. **(C)** U266 cells were treated with CSL (1 μM) for 0, 3, 6, and 12 h at 37°C. Whole-cell extracts were prepared, separated on SDS-PAGE, and subjected to Western blot analysis using antibodies against CXCR4 and MMP-9. The same blots were stripped and reprobed with β-actin antibody to show equal protein loading. The data shown is representative of at least two independent experiments.

Migration and invasion of myeloma cells play crucial role in the progression of myeloma (Siveen et al., 2014). We noted that CSL treatment can indeed inhibit the CXCL12-induced myeloma cell migration (**Figure 2A**). Using an *in vitro* invasion assay, we also found that CXCL12 significantly induced the invasion of U266 cells across the matrigel coated polycarbonate membrane and exposure to CSL significantly abrogated the invasive activity of U266 cells (**Figure 2B**). Since, the chemokine receptor CXCR4 and the key matrix metalloproteinase, MMP-9 are considered as important players in migration and invasion (Siveen et al., 2014), we analyzed if CSL has any effect on expression level of these two proteins. In our western blot analysis, we found out that treatment with CSL downregulated the expression of both the proteins in time-dependent manner, with maximal inhibition at 12 h (**Figure 2C**).

### CSL Augments the Apoptotic Effect of Bortezomib *in Vitro*

**FIGURE 3:**
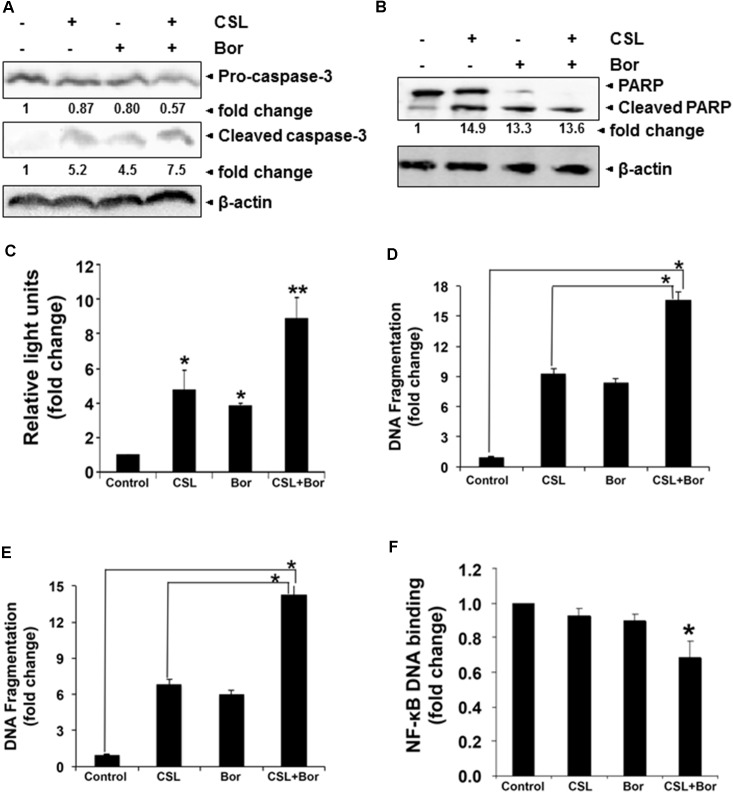
Celastrol potentiates the apoptotic effect of bortezomib in various MM cell lines. **(A)** U266 cells were treated with CSL (0.5 μM), bortezomib (10 nM) and a combination of both for 24 h at 37°C. Whole-cell extracts were prepared, separated on SDS-PAGE, and subjected to Western blot analysis using antibody against pro- and cleaved caspase-3. The same blots were stripped and reprobed with β-actin antibody to show equal protein loading. **(B)** U266 cells were treated with CSL (0.5 μM), bortezomib (10 nM) and a combination of both for 24 h at 37°C. Whole-cell extracts were prepared, separated on SDS-PAGE, and subjected to Western blot analysis using antibody against PARP. The same blots were stripped and reprobed with β-actin antibody to show equal protein loading. Results typical of two independent experiments are shown. **(C)** U266 cells were treated with CSL (0.5 μM), bortezomib (10 nM), and a combination of both for 24 h at 37°C. Caspase-3 activity/luminescence of each sample was measured in a plate-reading luminometer. Columns, mean; bars, SD. ^∗^*p* < 0.05, ^∗∗^*p* < 0.01. **(D)** H929 cells were treated with CSL (0.5 μM), bortezomib (10 nM), and a combination of both for 24 h at 37°C. Apoptosis was determined by measuring the degree of DNA fragmentation in the cytoplasm of cells. Columns, mean; bars, SD. ^∗^*p* < 0.05. **(E)** KMS-11 cells were treated with CSL (0.5 μM), bortezomib (10 nM), and a combination of both for 24 h at 37°C. Apoptosis was determined by measuring the degree of DNA fragmentation in the cytoplasm of cells. Columns, mean; bars, SD. ^∗^*p* < 0.05. **(F)** U266 cells were treated with CSL (0.5 μM), bortezomib (10 nM), and a combination of both for 24 h at 37°C. Twenty micrograms of the nuclear protein was used for DNA-binding assay as described in the section “Materials and Methods.” Columns, mean; bars, SD. ^∗^*p* < 0.05.

Our previous study has clearly indicated that celastrol can synergistically induce apoptosis of cultured human myeloma cells in combination with bortezomib and thalidomide (Kannaiyan et al., 2011a). In order to further confirm that CSL augments bortezomib induced apoptosis in U266 myeloma cells, we performed western blot analysis for detecting caspase-3 as well as PARP expression and also measured caspase-3 activity by luminescent assay. PARP cleavage to form a 85 kDa fragment of PARP mediated by caspase-3 is often associated with apoptosis and has been served as one hallmark of apoptosis and caspase activation (Ningegowda et al., 2017). U266 cells treated with a combination of CSL and bortezomib had a substantial activation of the effector molecule pro-caspase-3, with a concomitant increase in cleaved form of caspase-3 (**Figure 3A**) and the activation of caspase-3 led to the cleavage of a 118 kDa PARP protein into an 85 kDa fragment (**Figure 3B**). Caspase-3 activity in cells treated with a combination of CSL and Bor was also found to be significantly higher than CSL or bortezomib alone treated cells (**Figure 3C**).

We further found that CSL in combination with bortezomib caused significant increase in DNA fragmentation level in two diverse MM cell lines (16-fold in H929 cells and 14-fold in KMS-11 cells) as compared to the cells treated with CSL or bortezomib alone (**Figures 3D,E**).

### CSL Augments the NF-κB DNA Binding Inhibitory Activity of Bortezomib

The pro-inflammatory transcription factor NF-κB regulates the expression of multiple genes that control proliferation and survival and this oncogenic pathway is constitutively active in MM as well as in other malignancies (Li and Sethi, 2010; Li et al., 2015). Since, CSL augments the apoptotic effect of bortezomib, we next analyzed if this is also accompanied by the augmentation of inhibition of NF-κB pathway by bortezomib. The levels of constitutive NF-κB activation in MM cells were analyzed using ELISA-based TransAM NF-κB assay kit. The DNA-binding assay for NF-κB in nuclear extracts showed that CSL in combination with bortezomib reduced DNA binding activity of NF-κB, while CSL alone and bortezomib at sub-optimal doses alone had minimal effect on NF-κB activation (**Figure 3F**).

### CSL Augments Bortezomib Induced Inhibition of Tumor Growth in MM Xenograft Model

**FIGURE 4:**
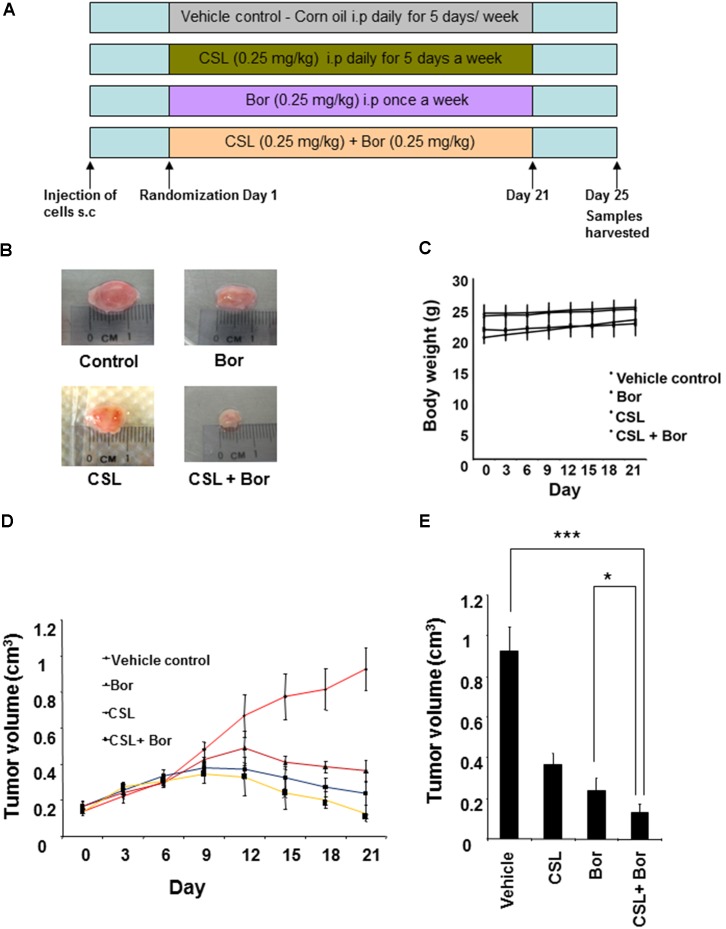
Celastrol potentiates the anti-tumor activity of bortezomib in xenograft mouse model. **(A)** A schematic representation of experimental protocol as described in the section “Materials and Methods.” Group I (control) received corn oil 100 μl i.p. for 5 days a week, group II received 0.25 mg/kg celastrol in 100 μl corn oil for 5 days a week, group III received 0.25 mg/kg bortezomib in 100 μl corn oil i.p. weekly, and group IV received 0.25 mg/kg celastrol in 100 μl corn oil i.p. 5 days a week and 0.25 mg/kg bortezomib in 100 μl corn oil i.p. weekly for three consecutive weeks. **(B)** Photographs of tumor tissue dissected from the mice after the end of treatment period. **(C)** The body weight of mice that was measured twice a week during the duration of the experiment. **(D)** Tumor size of each mice was measured using a vernier calipers twice a week for the duration of the experiment and tumor volume calculated using the formula [*L* ×*W*^2^]/2, where *W* and *L* are the width (short diameter) and the length (long diameter) of the tumor. **(E)** Tumor volumes in mice measured on the last day of the experiment with vernier calipers and calculated using the formula [*L* × *W*^2^]/2. Columns, mean; bars, SD. ^∗^*p* < 0.05, ^∗∗∗^*p* < 0.001.

We next analyzed whether CSL can augment the growth inhibition of bortezomib in human MM, U266 xenograft in nude mice. The detailed experimental procedure for this study has been depicted in **Figure 4A**. The results show that CSL alone induced significant inhibition of tumor growth compared with the corn oil treated controls (**Figures 4B,D**). CSL also augmented bortezomib induced inhibition of tumor growth in statistically significant manner (**Figure 4E**). Moreover, we did not notice any obvious side effects from the administration of CSL. There was actually no statistically significant weight loss in CSL treated groups either when used alone or in combination (**Figure 4C**).

### CSL Decreases the Serum Level of TNF-α and IL-6 in MM Tumor Bearing Mice

**FIGURE 5:**
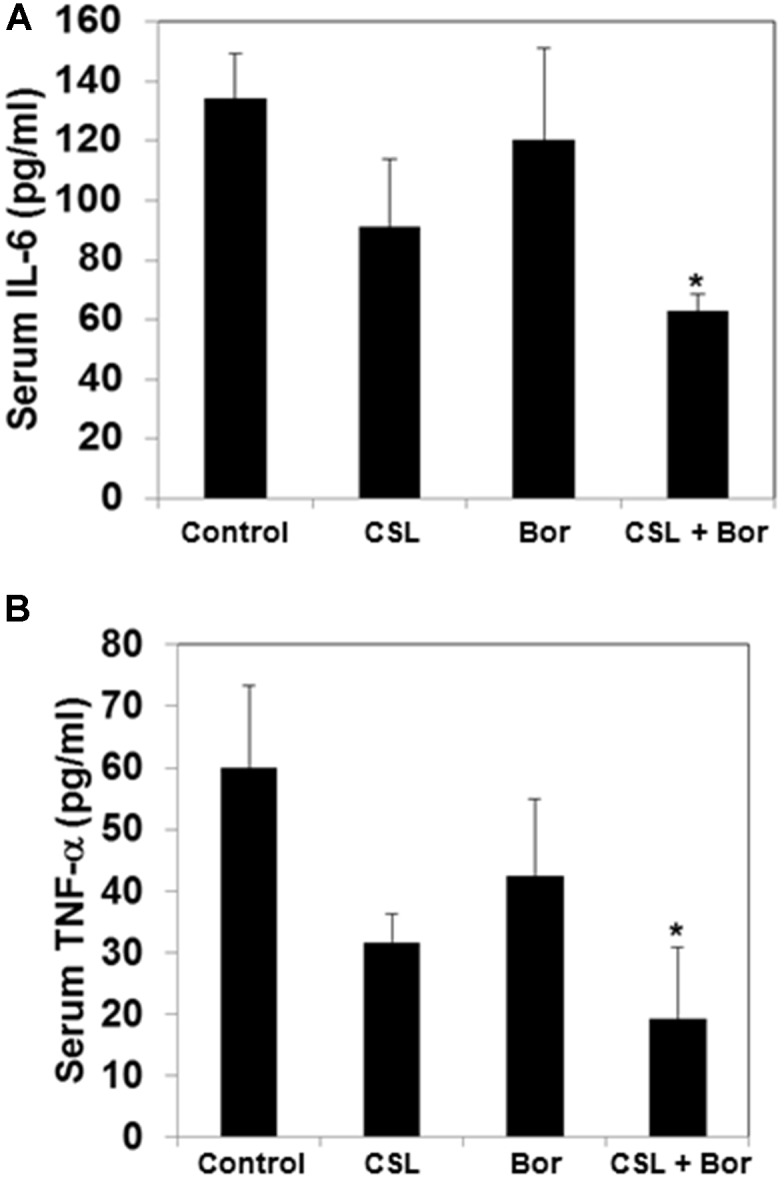
Celastrol modulates serum levels of IL-6 and TNF-α in MM tumor bearing mice. **(A)** All four groups of mice were treated as described in the section “Materials and Methods.” Sandwich ELISA assay was performed as per manufacturers’ instruction R&D systems (Minneapolis, MN, United States) to determine the levels of IL-6. ^∗^*p* < 0.05. **(B)** All four groups of mice treated as described in the section “Materials and Methods.” Sandwich ELISA assay was performed as per manufacturers’ instruction R&D systems (Minneapolis, MN, United States) to determine the levels of TNF-α. ^∗^*p* < 0.05.

The cytokines IL-6 and TNF-α have been reported to be involved in the progression myeloma and hence the effect of CSL and bortezomib on the levels of these two cytokines in serum samples obtained from mice was analyzed using an ELISA kit. The serum levels of both IL-6 and TNF-α level was found to be substantially higher in untreated tumor bearing mice whereas both CSL and bortezomib treated group had decreased levels of IL-6 and TNF-α as compared to control group (**Figures 5A,B**). The combination treated group of mice displayed statistically significant decrease in the serum level IL-6 and TNF-α.

## Discussion

The goal of this study was to analyze whether CSL can potentiate the apoptotic effects of bortezomib in MM cell lines and in a xenograft mouse model. We found that CSL not only significantly attenuated the proliferation of CD138^+^ plasma cells but also induced apoptosis in these cells. CSL was found to inhibit the migration and invasion of MM cell lines through downregulation of expression of MMP-9 and CXCR4 proteins. CSL augmented the apoptotic effects of bortezomib as observed by the caspase-3 activation and PARP cleavage. We further noted that CSL in combination with bortezomib effectively suppressed the growth of MM cells in a xenograft mouse model.

Migration and invasion are characteristic hall marks of tumor cells and both of these processes were found to be significantly downregulated upon CSL treatment.

Matrix metalloproteinases (MMPs), which are zinc-dependent endopeptidases, have been reported to regulate both cellular invasion and metastasis in malignant cells (Poudel et al., 2014) and the treatment of MM cells with CSL was also found to substantially reduce the protein levels of MMP-9 in a time-dependent manner. The role of CXCL12/CXCR4 axis not only plays an important role in the recruitment of hematopoietic progenitor cells but can also regulate the expression of various growth factors and cytokines that control the various hall marks of malignant cells (Rettig et al., 2012; Asri et al., 2016). Moreover, the expression of CXCR4 can also be regulated by activation of NF-κB and STAT3 transcription factors (Li et al., 2015; Chai et al., 2016). The above mentioned evidences prompted us to analyze whether CSL has any effect on the level of CXCR4 protein. We found that CSL substantially downregulates the CXCR4 expression in a time-dependent manner, in addition to its inhibitory effect on MMP-9 protein. Thus, CSL induced downregulation of CXCR4 and MMP-9 may explain the underlying mechanism behind the ability of CSL to inhibit the CXCL12 induced migration and invasion of MM cells and can also be attributed to the downregulation of NF-κB activation by CSL as reported previously by our group (Kannaiyan et al., 2011a).

Bortezomib is one of the major drugs in the armamentarium of myeloma, but it has some major limiting factors associated with its application including development of severe side effects and chemoresistance (Cavo, 2006). Interestingly, we have previously reported that CSL can synergistically induce the apoptosis of cultured human myeloma cells in combination with bortezomib (Kannaiyan et al., 2011a). In the current study, we have further demonstrated that CSL can also augment the apoptotic effects of bortezomib as demonstrated by western blot analysis for caspase-3 and PARP as well as caspase activity assay.

Bortezomib, a clinically approved proteasome inhibitor has been reported to suppress NF-κB pathway in MM by various mechanisms, such as abrogating degradation and causing accumulation of IκB, thereby suppressing transcriptional activation of NF-κB (Mitsiades et al., 2002; Markovina et al., 2008). Moreover, inhibition of NF-κB pathway by bortezomib is considered to be as one of major factors for its anti-myeloma activity (Yang et al., 2008; Demchenko and Kuehl, 2010), although several other oncogenic signal transduction cascades such as STAT3/Akt and ERK kinase have also been reported to be modulated upon bortezomib treatment (Mitsiades et al., 2002; Hideshima et al., 2003). CSL is also an effective proteasome inhibitor its potential effect on 26S proteasome activity may contribute to its reported pro-apoptotic activities against tumor cells (Yang et al., 2006; Dai et al., 2010). Moreover, our prior work has shown that CSL can inhibit the phosphorylation of p65 subunit, nuclear translocation of the p65 subunit and DNA binding ability of NF-κB in myeloma cells. It’s a well-known fact that when two drugs act synergistically, they may act in two different steps of the same pathway. So, we hypothesized that the observed combinatorial anticancer effects of CSL and bortezomib might translate from CSL’s ability to augment NF-κB inhibition by bortezomib. Indeed, the results of NF-κB DNA binding assay showed that CSL can enhance the NF-κB inhibition by bortezomib in statistically significant manner.

Multiple myeloma xenograft mouse model resembles the human myeloma better than the cultured cell lines. Though this model has the disadvantage of ignoring the role of bone marrow microenvironment and there are animal models that resemble the human myeloma better than xenograft model, this simple model was used to validate our *in vitro* findings (Ackler et al., 2010). So, we carried out the *in vivo* experiments to investigate if CSL can also augment the anti-myeloma activity of bortezomib *in vivo.* The results showed that CSL not only alone inhibited the tumor growth but it also augmented the bortezomib-induced abrogation of tumor growth in statistically significant manner. Moreover, no obvious side effects were noted in different groups of mice that were treated with CSL. We also found that that there was actually no minimal weight loss in CSL treated groups either when used alone or in combination.

NF-κB is one of the key proteins involved in the development of chemoresistance in myeloma and regulates the expression of various genes involved in proliferation, survival, and metastasis in myeloma (Li and Sethi, 2010; Li et al., 2015). TNF-α is one of the major pro-inflammatory cytokines secreted from myeloma cells and can stimulate bone marrow stromal cells to release IL-6, which can act as a potential growth factor for myeloma cells (Jurczyszyn et al., 2015; Mondello et al., 2017). Interestingly, the pharmacological blockers of TNF-α; namely thalidomide and its derivatives and bortezomib, have been reported to exhibit significant anti-myeloma activity (Demchenko and Kuehl, 2010; Fairfield et al., 2016). Interestingly, in our *in vivo* model, we found that there is a statistically significant reduction in the TNF-α levels in serum when both CSL and bortezomib were treated alone or in combination. We further analyzed the serum level of IL-6 in our *in vivo* model and found that there was a statistically significant reduction in the level of IL-6 either when the groups were treated with CSL and bortezomib alone or in combination.

In summary, we can conclude that CSL not only inhibits migration and invasion of myeloma cells but also can downregulate the expression of CXCR4 and MMP-9 proteins *in vitro*. CSL can also augment the bortezomib induced inhibition of NF-κB activation as well as potentiate the growth inhibition induced by bortezomib. However, xenograft MM models exclude the influence of bone marrow microenvironment on myeloma progression. So, these studies have to be replicated with orthotopic myeloma mouse models before proceeding with the detailed pharmacokinetic and toxicological studies with CSL for potential human use.

## Author Contributions

MS, JL, RK, NM, KM, and KS designed and performed the experiments. KA, AK, WC, and GS supervised the study and wrote the manuscript.

## Conflict of Interest Statement

The authors declare that the research was conducted in the absence of any commercial or financial relationships that could be construed as a potential conflict of interest.

## Abbreviations

Bcl-2: B cell lymphoma-2
Bcl-xL: Bcl-2 like protein 1
COX 2: cyclooxygenase 2
CXCL12: chemokine (C-X-C motif) ligand 12
CXCR4: C-X-C chemokine receptor type 4
EDTA: ethylenediaminetetraacetic acid
ELISA: enzyme-linked immunosorbent assay
FBS: fetal bovine serum
IL-6: interleukin 6
Mcl-1: induced myeloid leukemia cell differentiation protein Mcl-1
MM: multiple myeloma
MMP-9: matrix metalloproteinase-9
MOE: molecular operating environment
NF-κB: nuclear factor kappa-light-chain-enhancer of activated B cells
ROS: reactive oxygen species
SDS-PAGE: sodium dodecyl sulfate polyacrylamide gel electrophoresis
STAT3: signal transducer and activator of transcription 3
TNF-α: tumor necrosis factor-α
TNF-R2: tumor necrosis factor receptor 2
VEGF: vascular endothelial growth factor
VLA-4: very late antigen-4
